# The stepwise evolution of the exome during acquisition of docetaxel resistance in breast cancer cells

**DOI:** 10.1186/s12864-016-2749-4

**Published:** 2016-06-09

**Authors:** Stine Ninel Hansen, Natasja Spring Ehlers, Shida Zhu, Mathilde Borg Houlberg Thomsen, Rikke Linnemann Nielsen, Dongbing Liu, Guangbiao Wang, Yong Hou, Xiuqing Zhang, Xun Xu, Lars Bolund, Huanming Yang, Jun Wang, Jose Moreira, Henrik J Ditzel, Nils Brünner, Anne-Sofie Schrohl, Jan Stenvang, Ramneek Gupta

**Affiliations:** Sino Danish Breast Cancer Research Center, Copenhagen, Denmark; Center for Biological Sequence Analysis, Department of Systems Biology, Technical University of Denmark, Kemitorvet building 208, DK-2800 Lyngby, Denmark; Faculty of Health and Medical Sciences, Department of Veterinary Disease Biology, Section for Molecular Disease Biology, University of Copenhagen, Strandboulevarden 49, DK-2100 Copenhagen, Denmark; BGI-Shenzhen, Beishan Industrial Zone, Yantian District, Shenzhen, 518083 China; Department of Molecular Medicine, Aarhus University Hospital, Brendstrupgaardsvej 100, DK-8200 Aarhus N, Denmark; Department of Biomedicine, Aarhus University, Bartholins Allé 6, DK-8000 Aarhus C, Denmark; Department of Biology, University of Copenhagen, Ole Maaløes Vej 5, DK-2200 Copenhagen, Denmark; Princess Al Jawhara Albrahim Center of Excellence in the Research of Hereditary Disorders, King Abdulaziz University, Jeddah, Saudi Arabia; Macau University of Science and Technology, Avenida Wai long, Taipa, Macau, 999078 China; Department of Medicine and State Key Laboratory of Pharmaceutical Biotechnology, University of Hong Kong, 21 Sassoon Road, Pokfulam, Hong Kong; Department of Cancer and Inflammation Research, Institute of Molecular Medicine, University of Southern Denmark, J.B. Winsloews Vej 25, DK-5000 Odense, Denmark; Department of Oncology, Odense University Hospital, Sdr. Boulevard 29, DK-5000 Odense, Denmark

**Keywords:** Breast cancer, Docetaxel resistance, Taxane, Exome sequencing

## Abstract

**Background:**

Resistance to taxane-based therapy in breast cancer patients is a major clinical problem that may be addressed through insight of the genomic alterations leading to taxane resistance in breast cancer cells. In the current study we used whole exome sequencing to discover somatic genomic alterations, evolving across evolutionary stages during the acquisition of docetaxel resistance in breast cancer cell lines.

**Results:**

Two human breast cancer in vitro models (MCF-7 and MDA-MB-231) of the step-wise acquisition of docetaxel resistance were developed by exposing cells to 18 gradually increasing concentrations of docetaxel. Whole exome sequencing performed at five successive stages during this process was used to identify single point mutational events, insertions/deletions and copy number alterations associated with the acquisition of docetaxel resistance. Acquired coding variation undergoing positive selection and harboring characteristics likely to be functional were further prioritized using network-based approaches.

A number of genomic changes were found to be undergoing evolutionary selection, some of which were likely to be functional. Of the five stages of progression toward resistance, most resistance relevant genomic variation appeared to arise midway towards fully resistant cells corresponding to passage 31 (5 nM docetaxel) for MDA-MB-231 and passage 16 (1.2 nM docetaxel) for MCF-7, and where the cells also exhibited a period of reduced growth rate or arrest, respectively. MCF-7 cell acquired several copy number gains on chromosome 7, including ABC transporter genes, including *ABCB1* and *ABCB4*, as well as *DMTF1, CLDN12, CROT*, and *SRI*. For MDA-MB-231 numerous copy number losses on chromosome X involving more than 30 genes was observed. Of these genes, *CASK, POLA1, PRDX4, MED14* and *PIGA* were highly prioritized by the applied network-based gene ranking approach. At higher docetaxel concentration MCF-7 subclones exhibited a copy number loss in *E2F4*, and the gene encoding this important transcription factor was down-regulated in MCF-7 resistant cells.

**Conclusions:**

Our study of the evolution of acquired docetaxel resistance identified several genomic changes that might explain development of docetaxel resistance. Interestingly, the most relevant resistance-associated changes appeared to originate midway through the evolution towards fully resistant cell lines. Our data suggest that no single genomic event sufficiently predicts resistance to docetaxel, but require genomic alterations affecting multiple pathways that in concert establish the final resistance stage.

**Electronic supplementary material:**

The online version of this article (doi:10.1186/s12864-016-2749-4) contains supplementary material, which is available to authorized users.

## Background

Resistance to taxane therapy in breast cancer patients remains a major clinical problem with approximately 20 % of neo-adjuvant or adjuvant taxane treated patients experiencing disease recurrence with subsequent death from breast cancer. We here hypothesize that characterization of taxane resistance-associated molecular alterations and identification and validation of putative predictive markers will have the potential to result in more effective use of taxanes, with improved survival and reduced toxicities and costs. Currently, only a few predictive biomarkers are used in the clinical management of breast cancer. Among them is the estrogen receptor, used to predict sensitivity to endocrine therapy, and HER2 expression, used to predict sensitivity to HER2-targeting drugs, e.g. Trastuzumab (Herceptin) and Lapatinib. More recently, TOP2A gene aberrations were related to anthracycline sensitivity [1, 2] and two recent meta-analyses concluded that TOP2A aberrations predict an incremental benefit from anthracyclines [3, 4].

Taxanes (docetaxel, paclitaxel) are mitotic inhibitors and play a key role in the treatment of primary and advanced breast cancer [5–8]. Taxanes induce a mitotic block by binding to the beta-tubulin subunit of the microtubules, thereby preventing their disassembly [9]. However, resistance to taxanes may appear during or after taxane therapy, leading to increased patient morbidity and mortality. Prior studies in taxane resistance have particularly focused on expression of the *ABCB1* product permeability-glycoprotein (Pgp), which belongs to the superfamily of ATP-binding cassette (ABC) transporters [10]. Pgp is a xenobiotic drug efflux pump, and its overexpression has been extensively investigated as a predictor of multidrug resistance (MDR) to chemotherapeutics including taxanes [10, 11]. A meta-analysis of breast cancer, including 31 studies (>1200 patients), reported that approximately 40 % of all breast tumors expressed Pgp and Pgp expressing tumors were three times more likely to be chemotherapy-insensitive [11]. In addition to Pgp, several other ABC transporters reportedly confer an MDR phenotype [10], but an understanding of the mechanisms underlying the development of resistance to taxane remains incomplete.

Recently, analyses of the whole genome of breast tumors have been presented. Applying next-generation sequencing techniques, new breast cancer-related genes have been suggested [12, 13], and data obtained from various experimental platforms (DNA, RNA, protein) have been combined in an attempt to create integrated molecular characterizations of breast cancers [14]. In addition, several studies have successfully applied next generation sequencing to discover novel mechanisms of cancer chemotherapy resistance, and to elucidate tumor cell progression and survival properties during chemotherapy exposure [15–19]. To date, limited genomic alterations characterizing the development of taxane-resistant cancer cells have been identified. Here, we applied whole exome sequencing to in vitro breast cancer models of docetaxel resistance to acquire insight into resistance-related genomic changes and the process of resistance development. We sequenced the exome of two breast cancer cell lines (MCF-7 and MDA-MB-231) and their resistant sub-lines, which were isolated during several steps of successive development of resistance. We hypothesize that studying this evolution of docetaxel resistance will reveal genomic events that play important roles for the development of a docetaxel-resistant phenotype. Eventually, some of these mutations, either individually or as a panel, may potentially serve as predictive biomarkers of taxane therapy.

## Methods

### Cell lines

Resistant breast cancer cell lines were developed as previously described [20]. In brief, resistant breast cancer cells were developed by exposing cells to gradually increasing concentrations of docetaxel (Sanofi-Aventis, Hoersholm, Denmark) [20]. Cells were grown in complete medium (Dulbecco’s modified Eagle’s medium (DMEM) including L-glutamine, supplemented with 5 % fetal calf serum (FCS) as well as 1 % non-essential amino acids for the MCF-7 cells and 10 % FCS for the MDA-MB-231 cells; all obtained from Life Technologies, Carlsbad, USA) in a humidified atmosphere containing 5 % CO_2_ at 37 °C. For maintenance of resistant cells, docetaxel (MCF-7: 65 nM; MDA-MB-231: 150 nM) was added to the complete medium. Cell line identity was verified by the IdentiCell Cell Line Authentication method (Aarhus University Hospital, Denmark). The parental cell lines (MCF-7_PAR_ and MDA_PAR_), four sub-lines (MCF-7_SUB_ and MDA_SUB_) isolated from each of the two cell lines during development of resistance, and the final resistant cell lines (MCF-7_RES-65nM_ and MDA_RES-150nM_) were further characterized (Tables 1 and 2).

**Table 1:** Evolution of docetaxel resistance in MCF-7 cells

Docetaxel concentration	Passage numbers	Days at given concentration^a^	Subpopulation for analysis	Doubling time (Days)
0 (Parental cells)	-	-	MCF-7_PAR_	1.3
10 pM	1–3	12		
30 pM	4–6	13		
90 pM	7–9	18		
270 pM	10–12	17	MCF-7_SUB-0.27nM_	1.9
810 pM	13–15	12		
**1.2 nM**	**16–18**	**69**	MCF-7_SUB-1.2nM_	**1.8**
**1.8 nM**	**19–21**	**25**		
2.25 nM	22–24	13		
2.8 nM	25–27	19		
3.5 nM	28–30	20		
5 nM	31–33	18		
7.5 nM	34–36	12	MCF-7_SUB-7.5nM_	1.9
15 nM	37–39	13		
30 nM	40–42	16		
**45 nM**	**43–45**	**24**	MCF-7_SUB-45nM_	1.8
**55 nM**	**46–48**	**26**		
65 nM	49-		MCF-7_RES-65nM_	1.4

^a^Numbers in bold indicate concentration levels at which the cells took more than the average 20 days per concentration step to recover and go through 3 passages

**Table 2:** Evolution of docetaxel resistance in MDA-MB-231 cells

Docetaxel concentration	Passage numbers	Days at given concentration^a^	Subpopulation for analysis	Doubling time (Days)
Parental	-	-	MDA_PAR_	1.1
10 pM	1–3	16		
30 pM	4–6	13		
90 pM	7–9	18		
270 pM	10–12	14	MDA_SUB-0.27nM_	1.4
810 pM	13–15	20		
1.2 nM	16–18	14		
**1.8 nM**	**19–21**	**23**		
2.25 nM	22–24	16		
2.8 nM	25–27	13		
3.5 nM	28–30	18		
**5 nM**	**31–33**	**22**	MDA_SUB-5nM_	1.6
**15 nM**	**34–36**	**35**	MDA_SUB-15nM_	2.8
**45 nM**	**37–39**	**25**		
**60 nM**	**40–42**	**28**		
**80 nM**	**43–45**	**25**		
100 nM	46–48	18	MDA_SUB-100nM_	1.0
**120 nM**	**49–51**	**26**		
150 nM	52-		MDA_RES.150nM_	1.0

^a^Numbers in bold indicate concentration levels at which the cells took more than the average 20 days per concentration step to recover and go through 3 passages

### Characterization of cell lines

Docetaxel cytotoxicity was assessed using tetrazolium-based semiautomated colorimetric (MTT) assay as previously described [21]. Cells were plated and exposed to docetaxel as previously described [20]. Cell viability was calculated in percent compared to untreated control cells. A minimum of three independent experiments was performed for each of the parental cell lines, sub-lines and final resistant cell lines.

Stability of the resistant phenotype of the final resistant cell lines was investigated by MTT assay following one month of docetaxel withdrawal.

Cell growth was investigated by crystal violet assay. Briefly, on day 0, cells were seeded in 96 well microtiter plates (identical assays of 7000 and 10 000 cells/well, respectively) and one plate per day was stained on days 2–7; the assay was then performed as previously described [20]. The doubling time was calculated for cells in exponential growth phase using the equation N = N_0_ x e^kt^ assuming proportionality between absorbance and cell number. These analyses were conducted three times and the result from one representative experiment is reported.

Cell cycle was analyzed as follows: In 6 well plates 20E4 cells/well or 15E4 cells/well were seeded of the MCF-7 cell lines and MDA-MB-231 cell lines, respectively. Cells were cultured for 3 days in their standard growth medium supplemented with proper concentration of docetaxel for the resistant cell lines. Cell cycle analysis was performed using the Nucleocounter system NC-250^TM^ Two step cell cycle analysis kit (Chemometec, Allerød, Denmark). In brief, cells were washed in PBS and stained with 250 μl lysis buffer containing DAPI (10 μg/ml) for 5 min at 37° and thoroughly resuspended. Cells were stabilized by adding 500 μl stabilization buffer and cellular fluorescence quantified. Cell cycle distribution was calculated using the NC-250^TM^ Nucleoview software. These analyses were conducted three times and the result from one representative experiment is reported.

### DNA purification and sequencing

DNA was purified using Qiagen DNeasy Blood & Tissue Kit (Product #69604, Qiagen Nordic, Copenhagen, Denmark) according to the manufacturer’s instructions. The purity of the DNA was analyzed using a Nanodrop^TM^ spectrophotometer.

### Library construction and whole exome sequencing

Extracted DNA that passed quality control (undegraded, quantity > 3 μg) was selected for library construction. Agilent SureSelect Human All Exon Kit 50 Mb (Agilent Technologies, Santa Clara, USA) was used for exome enrichment and generated Illumina sequencing libraries according to the manufacturer’s protocols (Illumina, San Diego, USA). 1 to 2 μg of genomic DNA was sheared into 200 nt fragments on average by the Covaris S2 system (Covaris Inc, MA, USA), after which the DNA fragments were subsequently end-repaired, extended with an ‘A’ base on the 3′ end, ligated with indexing-specific paired-end adaptors followed by pre-capture PCR. Prepared libraries were hybridized for 24 h with biotinylated oligo RNA baits, and enriched with streptavidin-coated magnetic beads. Cluster generations of the libraries were then performed on Illumina cBot following the manufacturer’s recommended protocols (TruSeq PE Cluster Kit v3; Illumina). Finally, the clustered libraries were transferred onto HiSeq 2000 sequencers (Illumina) for 2 × 90 nt paired-end sequencing.

### Pre-processing of sequencing reads

Sequencing read quality was inspected using the FastQC software [22]. Adapter removal and read trimming were performed using Trimmomatic [23]. Sequencing reads were trimmed from the end (base quality less than Q20) and filtered by length (less than 25). Reads were mapped and aligned against the reference genome (build GRCh37) using Novoalign version 2.08.02 from Novocraft [24]. The alignment was sorted and then filtered to obtain a minimum mapping quality of Q30. This was performed using Samtools version 0.1.13 [25]. Reads mapping off target were removed with BedTools version 2.11.2 [26]. Duplicate reads were then removed using MarkDuplicates.jar from Picard’s suite of tools version 1.66 [27] and indexed using Samtools version 0.1.13. A local realignment around insertions and deletions was performed followed by a base quality score recalibration. Both steps were performed using the GATK Toolbox version 2.4-9 [28]. Finally GATK was used to left align insertions/deletions in the filtered, realigned and base quality score recalibrated BAM files.

### Variant detection, annotation and functional impact predictions

We applied a multi-sample calling strategy using UnifiedGenotyper [29] to call single nucleotide variation (SNVs), insertions and deletions. Raw SNV calls were filtered using variant quality score recalibration, whereas a basic filtering approach was employed on insertions and deletions.

Copy number alterations (CNAs) were identified using CONTRA (Copy Number Targeted Resequencing Analysis, [30]), - a tool specifically designed to detect CNAs from targeted re-sequencing data, ranging from smaller regions to whole-exome capture data. Copy number gains and losses in a region were estimated using base-level log-ratios and significance assigned using the null distribution of log-ratios as further detailed in [30]. CNAs were inferred for the resistant stages using the parental and any preceding stage as reference. We used Ensembl Variant Effect Predictor (version 2.8) for the annotation of detected mutations, and obtained information regarding the gene in which the mutation resides, the associated protein, and the protein domain potentially affected, among others. Also, SIFT [31] and PolyPhen [32] were used to assess how conserved the protein regions were in which the mutations occurred, indicating functional impact or possible impaired protein function. CNAs were annotated by querying Ensembl’s databases (v73), thereby providing information regarding genes overlapping a detected amplified or deleted region. In addition, information regarding previously obtained gene expression alterations, as described in [20], for final resistant cells was associated with identified CNAs.

### Variant classification

**Fig. 1:**
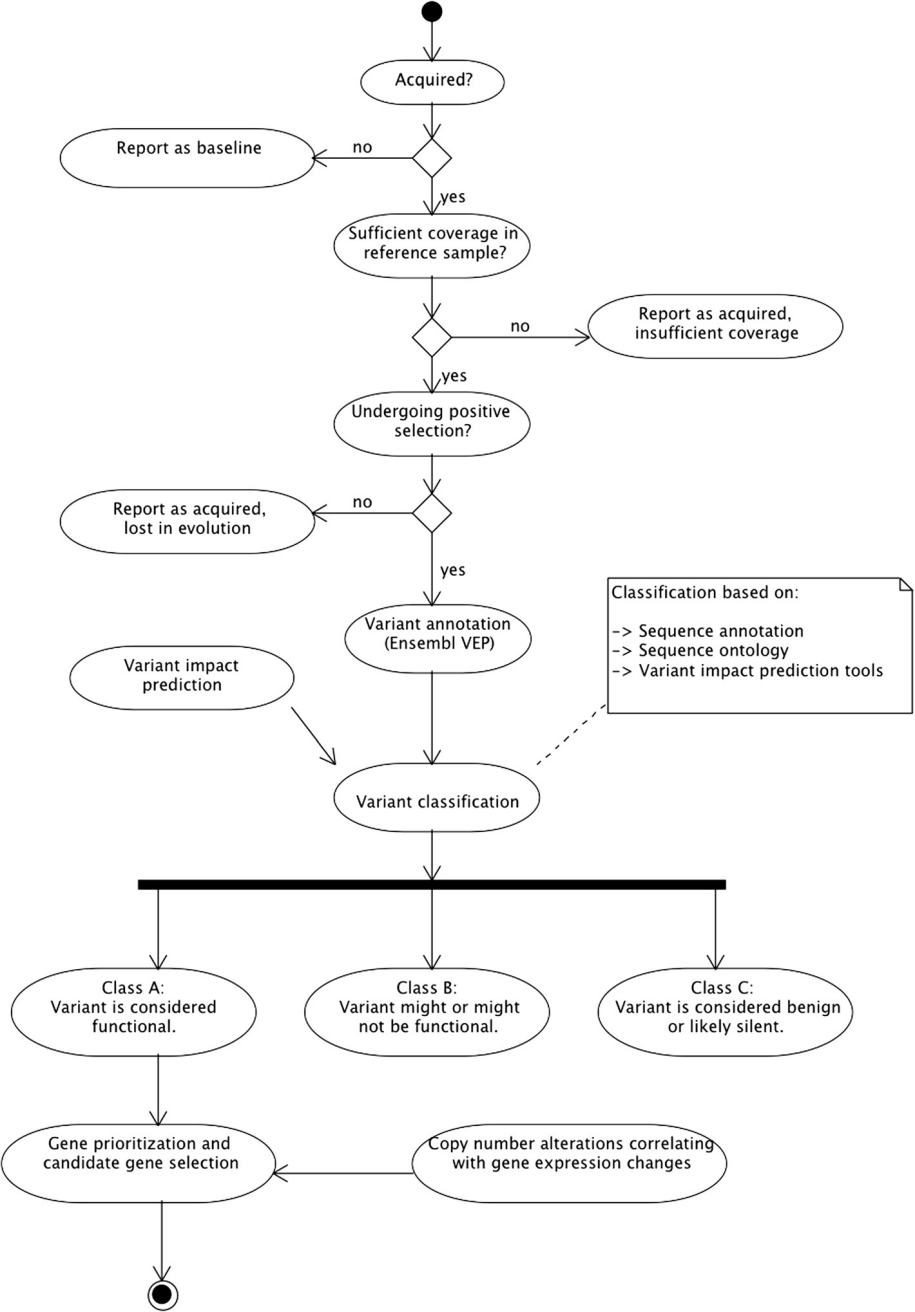
Workflow used in the interpretation and classification of detected mutations

An identified mutation was classified as acquired in a given resistant subline if it had not been called in the stage preceding it. Also, the preceding stage should have a read depth greater than four at the given position, and there should be no evidence of the alternative allele, i.e., the number of reads supporting the alternative allele should be zero. This allowed us to select mutations with confidence they were acquired in progressive stages and had not been seen before in assessable (sufficient sequence depth) regions. A similar approach was used for the CNAs. Because all CNAs are inferred with preceding stages as reference, they are per definition acquired. As a cell line progresses towards a fully resistant stage, it acquires and accumulates mutations and CNAs, but far from all variants are equally functionally important, and only a fraction of the variants are likely to play a role in the development of resistance to docetaxel. Several strategies were employed to narrow down the list of mutations (and genes) of interest for inclusion in further downstream analysis primarily focusing on the idea that variation under selection would provide a functional advantage to cell survival. A variant was inferred to be under selection if it was acquired in a given stage and then also seen in the following stages as well as in the final resistant stage. Mutation events that were not seen in subsequent stages were not considered in the analysis. Thus only accumulating events were considered, and such variants denoted as *acquired selective-events*. In addition, identified acquired selective-events were grouped into three classes according to their likely functional character: *Class A* variants were regarded as *likely-functional events* and included splice donor and splice acceptor variants, stop gain and stop loss variants, frameshift variants, initiator codon variants, transcription factor (TF) binding site variants, missense variants (if predicted to be deleterious by either SIFT and/or PolyPhen or if located in a protein domain). *Class B* variants encompassed events wherein the impact was more difficult to determine without further functional studies, and included inframe insertions and deletions, missense variation of predicted benign character, splice region variants, incomplete terminal codon variants, mature microRNA (miRNA) variants, nonsense-mediated decay (NMD) transcript variants and various types of regulatory variants. Finally, *Class C* variants contained those variants likely considered benign or ‘silent’, although we cannot completely exclude a potential functional impact of such variants on the protein product, but simply assume a functional character is less likely or would need significant additional evidence to be credible. This class included synonymous variants, stop retained variants, 5 prime UTR variants, 3 prime UTR variants, non-coding exon variants, nc-transcript variants, intron variants, upstream and downstream gene variants and intergenic variants. Figure 1 gives an overview of the classification procedure just described.

### Gene expression profiling

Gene expression analysis (microarrays) has previously been performed on the cell lines by methods described elsewhere [20], that included the parental and final resistant stages (MCF-7_PAR_/MCF-7_RES-65nM_ and MDA_PAR_/MDA_RES-150nM_). We correlated available gene expression profiles for these stages with identified copy numbers to search for CNAs that might explain changes observed in the expression of certain genes in the final resistant cells (MCF-7_RES-65nM_ and MDA_RES-150nM_).

### Relating selective events to cancer hallmarks

Cancer hallmarks [33] comprise a list of acquired biological changes defining cancer as such. Here, we used literature curation to categorize genes carrying a selective event according to the eleven cancer hallmark categories: Activating invasion and metastasis (H1), Enabling replicative immortality (H2), Evading growth suppressors (H3), Evading Immune destruction (H4), Genome instability and mutation (H5), Inducing angiogenesis (H6), Reprogramming energy metabolism (H7), Resisting cell death (H8), Sustaining proliferative signaling (H9), Tumor-promoting inflammation (H10) and Tumor microenvironment (H11).

For a subset of the detected variation, we assessed the association of the corresponding genes and the eleven cancer hallmarks. This subset included *Class A* and *B* variants plus CNA events that correlated with expression and included 916 genes in total, of which 396 had previously been associated with breast neoplasm, and these were then mapped to the hallmark classes.

### Random walk-based gene prioritization

Motivated by the assumption that potential new taxane resistance-associated genes might harbor characteristics similar to those already found to cause resistance to taxanes, we used a network-based gene prioritization strategy implemented in the Cytoscape plugin GPEC [34]. GPEC scores and ranks genes based on their functional similarity with known resistance genes identified by literature curation, as previously described [Ehlers et al, unpublished observations]. Only *Class A* mutations (see above) and CNAs that associate with a changed expression of the gene were further ranked with GPEC. We ranked genes in each stage as well as across all stages to identify the potentially most important genes for a given stage as well as the most important genes overall.

### Gene stage enrichment analysis

Online enrichment tools were applied in order to identify co-functioning genes in each stage with *Class A* mutations or CNAs and annotate these to biological functions. Genes in each stage of the MCF-7 and MDA-MB-231 cell lines were analysed using DAVID Bioinformatics Resources 6.7 [35, 36] and GOrilla [37, 38]. The resulting groups of genes identified by DAVID or GOrilla were only considered important if the enrichment score for a gene set was ≥ 1. Redundant GO terms were simplified using REVIGO [39] and AmiGO 2 [40]. Similar biological annotations were evaluated and redundant annotations were consolidated.

## Results and discussion

### In vitro model of docetaxel resistance

Two different human breast cancer cell lines, MCF-7 and MDA-MB-231, which are widely used as models for hormone-dependent and -independent human breast cancer, respectively, were cultured in the presence of increasing concentrations of docetaxel. The development process and investigated subpopulations are outlined in Tables 1 and 2. A total of 17 (MCF-7) and 18 concentrations (MDA-MB-231) were applied to the cells, and both cell lines spent an average of 20 days at each concentration step through 3 successive passages. For MCF-7, we observed temporary growth arrest at 1.2 nM docetaxel (69 days) followed by normal growth rate at concentrations above 1.2 nM. MDA-MB-231 cells grew slower at 15 nM docetaxel, but did not arrest growth at any time. For MCF-7, resistance development was stopped at 65 nM docetaxel (MCF-7_RES-65nM_), and for MDA-MB-231, the final concentration was 150 nM (MDA_RES-150nM_). MCF-7_RES-65nM_ and MDA_RES-150nM_, their respective parental cell lines and four additional sub-lines per cell line (MCF-7_SUB_ and MDA_SUB_), isolated during development of resistance, were further characterized.

### Docetaxel cytotoxicity

**Fig. 2:**
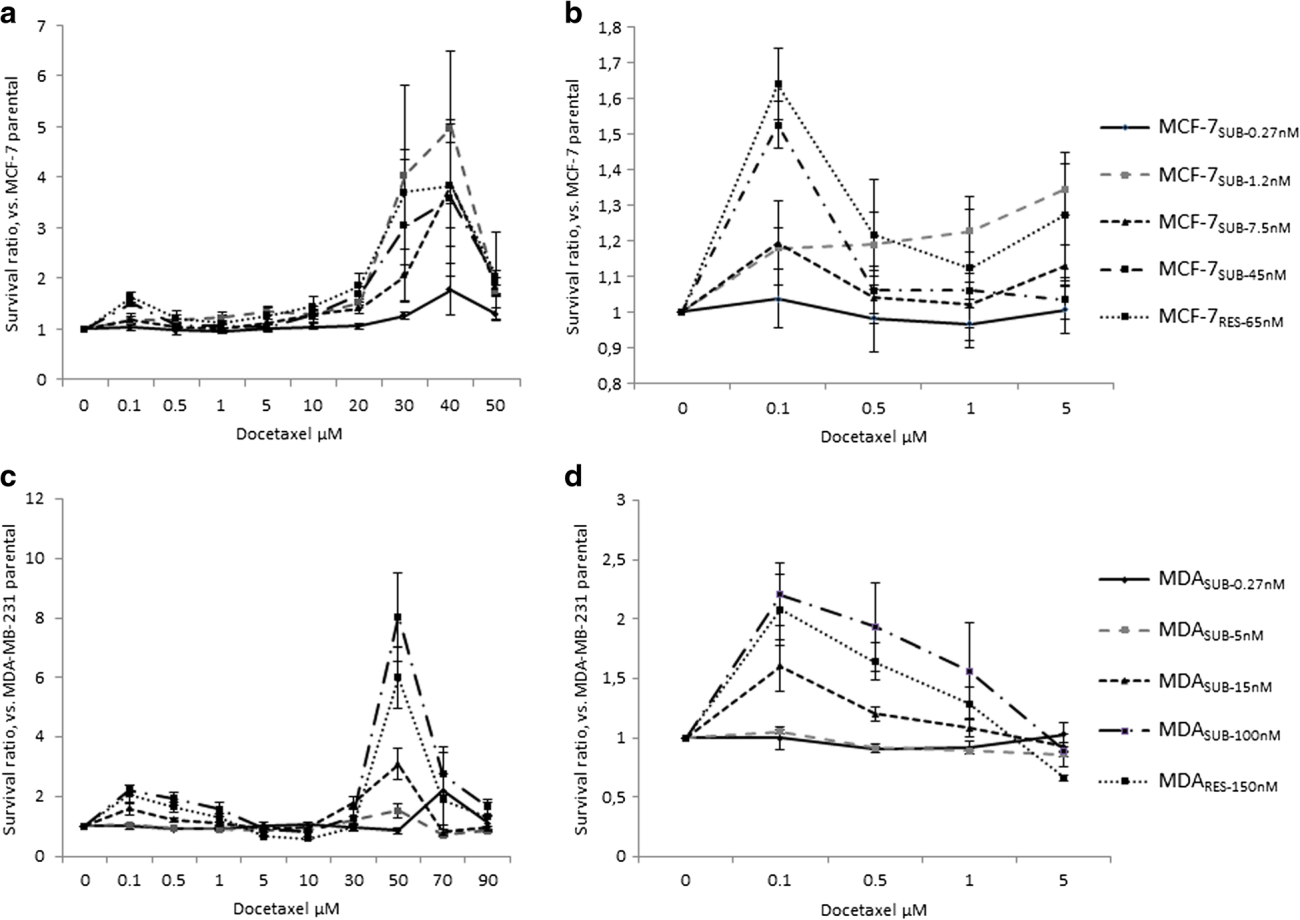
Sensitivity of parental and reistant sub lines to docetaxel. The survival ratio of MCF-7 (**a**, **b**) and MDA-MB-231 (**c**, **d**) sub-lines compared to corresponding parental cell lines (MCF-7_PAR_/ MDA_PAR_). Cells were exposed to docetaxel at the indicated concentrations for 72 h and survival was estimated using an MTT assay. Values are expressed as relative values compared to the parental cell lines. Mean values of three independent experiments ± SEM are shown

Culturing of cells with continuous exposure to increasing concentrations of docetaxel induced resistance to the drug. As previously described, both MCF-7 and MDA-MB-231 cells showed a biphasic response pattern when exposed to docetaxel, with their final resistant sub-lines being significantly more resistant both in a first phase at lower docetaxel concentrations, and in a second phase at higher concentrations [20]. The sensitivity of selected sub-lines isolated during development of resistance was investigated and the survival of each of the sub-lines was calculated as the ratio of surviving cells in each sub-line compared to the corresponding parental cell line. The results are depicted in Fig. 2a-d. In MCF-7, when exposing cells to high concentrations of docetaxel (20–50 μM) resistance was observed as early as in the first investigated sub-line (MCF-7_SUB-0.27nM_) as well as in the rest of the investigated cells (Fig. 2a). At lower concentrations of docetaxel (0.1–5 μM), however, only sub-lines that had been exposed to higher concentrations of the drug (MCF-7_SUB-45nM_ and MCF-7_RES-65nM_) were resistant compared to the parental MCF-7 cell line (Fig. 2b). In MDA-MB-231, resistance to docetaxel developed gradually as cells were exposed to increasing concentrations of the drug; this applies to both low and high concentrations of docetaxel (Fig. 2c and d). MDA_SUB-0.27nM_ did not differ from parental MDA-MB-231 cells with regard to docetaxel sensitivity (Fig. 2c and d).

Stability of the final resistant phenotype was confirmed as both MCF-7_RES-65nM_ and MDA_RES-150nM_ showed no change in sensitivity to docetaxel following one month of culturing without docetaxel exposure (data not shown). This suggests that the phenotypic alterations in sensitivity to docetaxel are due to genomic alterations.

### Growth pattern

The doubling times for parental MCF-7 and MDA-MB-231 cells plus the derived docetaxel-exposed cell lines are listed in Tables 1 and 2. For MCF-7, doubling times increased slightly as cells developed resistance to docetaxel. The reported doubling times apply to cells growing stably at the indicated concentrations. However, as previously noted, cells went through a period of arrested growth at 1.2 nM docetaxel. In docetaxel-treated MDA-MB-231 cells, doubling times first increased, but became similar to parental cells after MDA_SUB-15nM_.

### Cell cycle

**Fig. 3:**
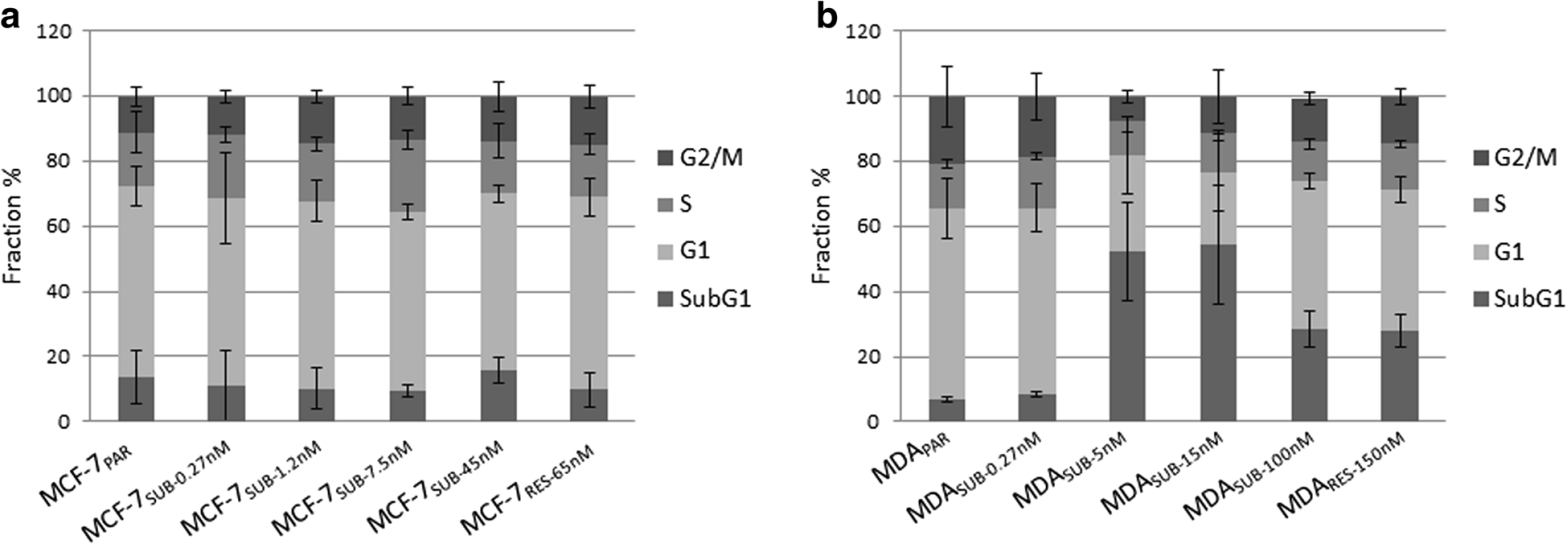
Cell cycle analysis of parental cells and resistant subclones. The bar charts show the distribution of cells in cell cycle phases G2/M, S, G1 and SubG1 for MCF-7_PAR_ and docetaxel resistant subclones (**a**) and MDA_PAR_ and resistant subclones (**b**). The resistant subclones were cultured in the presence of the indicated docetaxel concentrations. Mean values of three independent experiments ± SEM are shown

The distribution of parental and docetaxel-exposed MCF-7 sub-clones in the different phases of cell cycle was also examined (Fig. 3). MCF-7 parental cells and docetaxel-exposed MCF-7 sub-clones showed similar distributions, with the majority of the cells in G1 (Fig. 3a). For MDA-MB-231 cells, some clear differences in the fractions were observed when compared to the parental cells. For sub-lines MDA_SUB-5nM_ and MDA_SUB-15nM_ considerably fewer cells were in G1 and more cells were in subG1; this pattern, to a lesser degree, was also observed in MDA_SUB-100nM_ and MDA_SUB-150nM_ (Fig. 3b).

### The evolution of resistance – an orchestration of events

Between 55,000 and 59,000 single point mutations and 5,800–6,200 indels (Additional file 1: Table S1) were detected in each sub-line sequenced indicating a high burden or initial variation as compared to the human reference used in the study. The amount of acquired variation detected in each sub-line was variable and ranged from a few to just over 560 mutations, with most variants being acquired in the early phase of resistance development (MCF-7_SUB-0.27nM_ and MDA_SUB-0.27nM_) (Tables 3 and 4). Surprisingly, only a small percentage of variation acquired in a given cell sub-line was actually carried on to the final resistant stage (Tables 3 and 4). The majority of acquired genomic variants appeared to be silent (Tables 5 and 6), and only a small fraction thereof were classified as likely functional events (variant *Class A*, Fig. 1) (Additional file 1). A variable number of missense mutations were predicted to be deleterious by SIFT and Polyphen in each sub-line. In both cell lines, the sub-lines MCF-7_SUB-0.27nM_, MCF-7_SUB-1.2nM_, MDA_SUB-0.27nM_, and MDA_SUB-5nM_ accounted for the largest number of deleterious missense mutations in the category of acquired selective-events. Mapping of a selected number of genes to the cancer hallmark categories [33] revealed that most genes fall into the categories ‘sustaining proliferative signaling’ and ‘activating invasion and metastasis’ (Additional file 2). SIFT and Polyphen have been used as major determinants for change in function, and we believe that change in conserved sequence that these tools pick up are indicative both of loss and gain in function. However it has been shown in prior studies that these tools capture loss-of-function variants better than gain-of-function variants, and we would like to point out that some gain of function missense variants will be missed in the analysis.

**Table 3:** The number of acquired and selected variants detected in the MCF-7 sub-lines

	MCF-7	MCF-7	MCF-7	MCF-7	MCF-7
	SUB-0.27nM	SUB-1.2nM	SUB-7.5nM	SUB-45nM	RES-65nM
Acquired SNVs	506	179	140	181	253
Selected SNVs	166	84	27	50	
Acquired Indels	109	40	28	29	43
Selected Indels	31	11	5	14

**Table 4:** The number of acquired and selected variants detected in the MDA-MB-231 sub-lines

	MDA	MDA	MDA	MDA	MDA
	SUB-0.27nM	SUB-5nM	SUB-15nM	SUB-100nM	RES-150nM
Acquired SNVs	567	363	361	409	242
Selected SNVs	174	71	14	42	
Acquired Indels	103	28	31	23	34
Selected Indels	29	6	7	13

**Table 5:** Variant types: acquired and selected variants in MCF-7 sub-lines

Consequence	MCF-7	MCF-7	MCF-7	MCF-7	MCF-7
	SUB-0.27nM	SUB-1.2nM	SUB-7.5nM	SUB-45nM	RES-65nM
3 prime UTR variant	26	6	2	2	29
5 prime UTR variant	6	6	1	2	21
NMD transcript variant	70	33	3	14	128
TF binding site variant	0	0	0	0	0
Downstream gene variant	169	85	29	38	236
Feature elongation	29	2	8	22	20
Feature truncation	63	38	7	18	75
Frameshift variant	0	1	0	0	0
Inframe insertion	0	0	0	0	0
Intron variant	618	228	70	136	656
Mature miRNA variant	0	0	0	0	0
Missense variant	44	55	0	12	85
Nc transcript variant	182	89	12	58	203
Non coding exon variant	45	36	3	13	75
Regulatory region variant	27	13	5	12	54
Splice acceptor variant	0	0	0	0	12
Splice region variant	12	5	1	6	17
Stop gained	0	4	0	0	10
Synonymous variant	35	24	3	5	68
Upstream gene variant	95	66	13	38	195
Total	1421	691	157	376	1884

**Table 6:** Variant types: acquired and selected variants in MDA-MB-231 sub-lines

Consequence	MDA	MDA	MDA	MDA	MDA
	SUB-0.27nM	SUB-5nM	SUB-15nM	SUB-100nM	RES-150nM
3 prime UTR variant	25	8	1	3	28
5 prime UTR variant	6	7	1	0	14
NMD transcript variant	68	27	4	20	115
TF binding site variant	1	0	0	0	0
Downstream gene variant	197	97	14	40	361
Feature elongation	47	11	18	30	48
Feature truncation	59	14	0	13	29
Frameshift variant	0	0	0	1	0
Inframe insertion	1	0	0	0	0
Intron variant	741	224	51	97	769
Mature miRNA variant	0	0	0	1	0
Missense variant	18	43	4	9	130
Nc transcript variant	202	78	16	30	228
Non coding exon variant	57	37	7	5	74
Regulatory region variant	32	11	1	8	42
Splice acceptor variant	0	0	0	0	0
Splice region variant	22	15	1	2	45
Stop gained	0	2	0	0	9
Synonymous variant	60	30	0	16	61
Upstream gene variant	169	62	5	17	170
Total	1705	666	123	292	2123

In addition to detecting single mutational events, small insertion and deletions, we also used the exome data to identify CNAs acquired as a consequence of cell line development during docetaxel exposure. While copy number detection from whole exome sequencing data still requires further development, several methods have emerged [30, 41–44]. Here we used CONTRA [30] to identify regions of amplification or deletion in the cell line samples. After mapping CNA regions to genes, we then correlated CNA-genes detected in any sub-line with the expression profiles of the gene in the final resistant sub-lines (MCF-7_RES-65nM_/ MDA_RES-150nM_) compared to the parental cell lines (MCF-7_PAR_/ MDA_PAR_). This approach made it possible for us to reduce the amount of false negatives due to insufficient coverage. For instance, if a CNA event was detected in early stages of resistance development, but not found in later stages, despite the fact that the gene in which the CNA event was found was observed to be up-regulated in the final stage, this speaks in favor of the CNA still being present at this stage, but we are just not able to detect it with our exome data alone. The copy number profiles were very different between the two cell lines. Of primary interest for the MCF-7 cells were several copy number gains acquired during exposure to increasing concentrations of docetaxel, most of which resided on chromosome 7, but also on chromosome 4, 5, 8, 11 and 14. The MDA-MB-231 sub-lines predominately acquired copy number losses, primarily on the X chromosome, but also on chromosome 2, 6, 7, 9, 15 and 21, (Additional file 3).

It should be pointed out that despite the additional gene expression data, there are still inaccuracies in copy number analyses that will be expected. For instance, since many CNAs are smaller than the gene size, these likely result in alternative transcript isoforms rather than changing gene expression.

### Genomic alterations potentially driving the development of docetaxel resistance

To further rank and prioritize selected variants consisting of *Class A* variation and CNAs that correlated with expression profiles of the corresponding gene, we used a network-based approach [34] to measure the similarity of this gene set to genes previously associated with resistance to taxanes in the literature.

**Fig. 4:**
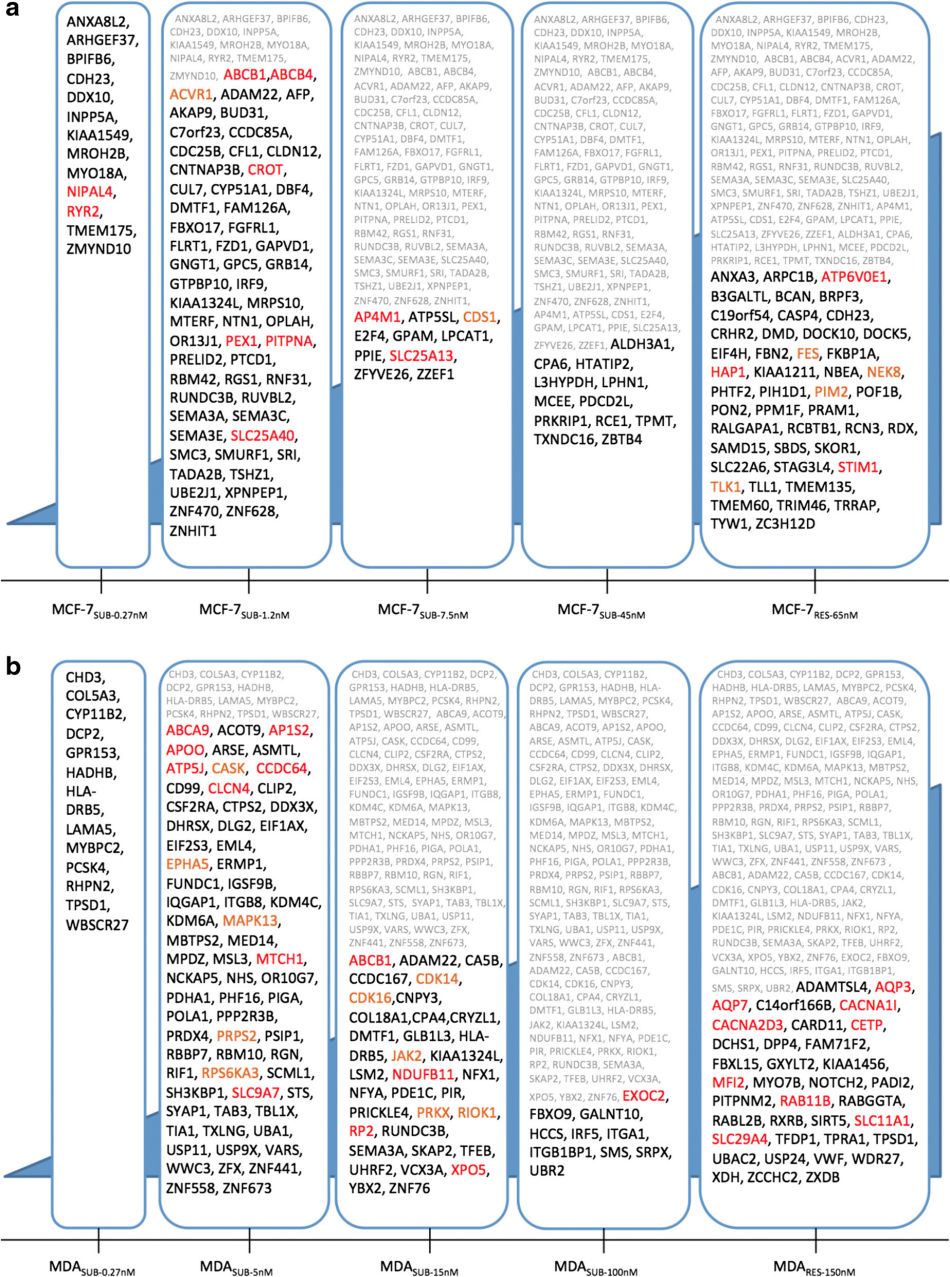
Genes with a *Class A* mutation or CNA in each stage in the MCF-7 (**a**) and MDA-MB-231 (**b**) cell lines. Genes involved in transport are marked in red, while kinases are marked in orange. Shaded grey are genes where mutations have accumulated from prior stages

For the network-based analysis performed across all stages, the top five ranked genes were ABCB4, CDC25B, FES, ACVR1 and RDX for the MCF-7 sub-lines, and JAK2, CARD11, KDM4C, VWF, CA5B for the MDA-MB-231 sub-lines (Additional file 4). A similar analysis performed on each sub-line separately revealed the genes potentially most important for resistance development in the sub-lines and resistant stage in MCF-7 (Fig. 4a) and MDA-MB-231 (Fig. 4b) (Additional files 4 and 5). The enrichment analyses of the genes in each developmental stage for the MCF-7 and MDA-MB-231 cell lines identified relevant groups of genes were annotated for MCF-7_SUB-1.2nM_ and MCF-7_RES-65nM_ (Table 7), while enriched groups in the MDA-MB-231 cell line were identified for MDA_SUB-5nM_ and MDA_RES-150nM_ (Table 8). Thus, at a general level genomic change occuring midways in the acquisition of docetaxel resistance and in the resistant stage of both cell lines were identified to be associated with various biological annotations. In the early phase of resistance development (MCF-7_SUB-0.27nM_ and MDA_SUB-0.27nM_) the acquisition of a number of deleterious mutations as well as a single CNA event in the MDA-MB-231 sub-line was observed. Of particular interest was the acquisition of a number of single point mutations in genes such as RYR2, DDX10, CDH23, ANXA8, CHD3, RHPN2 and HLA-DRB5 as well as a copy number loss in HADHB. These genes are observed to code for proteins having several interactions with proteins already associated with taxane resistance. The genes RYR2, CDH23 and RHPN2 are altered in both cell line model systems. While the RYR2 gene has acquired a deleterious point mutation in MCF-7_SUB-0.27nM_, it is observed to be down-regulated in MDA_RES-150nM_. The CDH23 gene is altered twice in the MCF-7 cell line in that a deleterious point mutation is acquired in early stage (MCF-7_SUB-0.27nM_) of resistance development followed by a copy number loss in the gene in the final resistant stage (MCF-7_RES-65nM_) where we also measured the gene as down-regulated. The gene is down-regulated in the MDA_RES-150nM_ as well. The CDH23 protein interacts with the product of ATP2B2, which have been associated with resistance to paclitaxel, where inhibition of ATP2B2 leads to increased sensitivity towards the action of paclitaxel [45]. Finally, RHPN2 is encoding the Rho GTPase binding protein 2 and interacts with the products of SFN and HNF4A, which have been associated with resistance to taxanes. A deleterious point mutation is observed in the RHPN2 gene in MDA_SUB-0.27nM_, while the same gene is observed to be down-regulated in MCF-7_RES-65nM_, strengthening the hypothesis of a role for this gene in the development of taxane resistance.

**Table 7:** Annotation of genes with *Class A* mutation or CNAs in MCF-7 sub-lines

MCF-7	MCF-7	MCF-7	MCF-7	MCF-7
SUB-0.27nM	SUB-1.2nM	SUB-7.5nM	SUB-45nM	RES-65nM
	Cell motility			Chromatin modification RCBTB1, BRPF3, TLK1, TRRAP
	CFL1, SEMA3C, NTN1, ACVR1			
	Negative regulationof signal transduction			
	RGS1, SMURF1, GRB14, ACVR1			
	Cell morphogenesis			
	CFL1, SEMA3A, NTN1			
	Neural crest cell development			
	CFL1, SEMA3C, ACVR1			
	Axon guidance			
	SEMA3E, CFL1, SEMA3C, SEMA3A, NTN1			
	Mitotic cell cycle process			
	DBF4, AKAP9, ABCB1, CUL7, CDC25B, ACVR1, CFL1

**Table 8:** Annotation of genes with *Class A* mutation or CNAs in MDA sub-lines

MDA	MDA	MDA	MDA	MDA
SUB-0.27nM	SUB-5nM	SUB-15nM	SUB-100nM	RES-150nM
	Chromatin modification	Nucleotide-binding		Protein dimerization activity
	MSL3, KDM6A, PHF16, KDM4C, RBBP7	ABCB1, JAK2, RIOK1, CDK16, CDK14, PRKX		NOTCH2, VWF, SLC11A1, DPP4
	Purine nucleoside binding, adenyl nucleotide binding, atp-binding			T cell activation
	EPHA5, RPS6KA3, ABCA9, DDX3X, UBA1, MAPK13, CASK, CTPS2, VARS, PRPS2, CLCN4			CARD11, SLC11A1, DPP4
	Kinase			Transport
	EPHA5, RPS6KA3, MAPK13, SH3KBP1, CASK, PRPS2			SLC11A1, CACNA1I, MFI2, CACNA2D3, AQP7, AQP3
	Regulation of transcription from RNA polymerase II promoter			
	CASK, MED14, RBBP7, TBL1X			
	Transport			
	CLCN4, CCDC64, AP1S2, CASK, ABCA9, ATP5J, APOO			

Midway through resistance development (MCF-7_SUB-1.2nM_ and MDA_SUB-5nM_), the acquisition of a number of CNAs was observed in both model systems. The MCF-7_SUB-1.2nM_ had acquired several copy number gains on chromosome 7, which involved the ABC transporters genes ABCB1 and ABCB4. The ABCB1 gene, also known as MDR1, encodes Pgp, and is to date the best characterized and understood chemotherapy efflux transporter [46, 47]. Pgp expression has been correlated with resistance to taxanes and has been extensively studied in breast carcinomas [11]. We recently confirmed that upregulation of MDR1 mRNA and the corresponding Pgp is a major resistance mechanism in docetaxel-resistant breast cancer cells in vitro [20]. Also of interest were copy number gains acquired in the genes DMTF1, CLDN12, CROT, and SRI, all located on chromosome 7. Though difficult to determine with our exome data, it is likely that a subset of these copy number gains are truly just one larger regional gain acquired on chromosome 7, which coincides with the temporary growth arrest observed in these cells as mentioned previously. Interestingly, upregulation of DMTF1, CLDN12, CROT, and SRI together with upregulation of other genes, such as ASK, CDK6, MGC4175 and MCM7, have previously been detected in MDR1-positive taxane-resistant ovarian cancer cell lines [48]. Amplification of this particular region on chromosome 7q, as seen in our study, has been seen in other studies and suggested as a fundamental mechanism of acquired resistance to taxanes [49]. In our study, this amplified region also included cell-cycle genes such as CDC25B, SMC3, AKAP9, and DBF4, as well as the unknown transmembrane protein C7orf23 (TMEM243). Overexpression of C7orf23 has been associated with resistance to paclitaxel [50]. Other observations made in the MCF-7_SUB-1.2nM_ cell line worth mentioning included a copy number gain in IRF9 as well as a copy number loss in CFL1. Overexpression of this IRF9 has been associated with resistance to paclitaxel [51] and a link between CFL1 expression and taxane resistance has been suggested [52]. In addition, CNAs were observed in several microtubule dynamic associated genes including gains of SEMA3E, ADAM22, AKAP9, SEMA3A and loss of SMURF1, NTN1, CFL1, and CDC25B, and a deleterious point mutation was detected in CUL7 which is also involved in microtubule dynamics [53]. In particular, the products of CFL1, CDC25B, and SMURF1 are highly interacting with proteins encoded by genes with a known association to taxane resistance.

In MDA_SUB-5nM_, a larger fraction of genes located on chromosome X acquired a copy number loss that was correlated with down-regulated genes in MDA_RES-150nM_. The copy number losses on chromosome X included 39 genes (Additional file 4), of which several were associated with development of taxane resistance by the network-based analysis. Interestingly, one of these genes, POLA1, has been identified as a microtubule-binding and -modulating protein [54], and three SNPs in PRDX4 have been associated with docetaxel clearance [55]. Among the 39 genes, not highly ranked, but still of interest due to their functional role of their protein products in the cell, were DDX3X, involved in chromosome segregation, TBL1X, involved in spindle microtubule, IQGAP1, involved in stabilization of the microtubules, PPP2R3B, involved in cell cycle arrest, and RPS6KA3, involved in the cell cycle. Although it is not possible to determine with our exome data, it is likely that the copy number losses found on chromosome X are actually due to the acquisition of one larger regional loss of the chromosome, an event that might be a key driver of the development of docetaxel resistance.

In sub-lines exposed to higher concentrations of docetaxel (7.5nM–15nM) a small number of single point mutations of *Class A* together with several CNAs were observed in the cells. In MCF-7_SUB-7.5nM_, copy number losses were observed in CDS1, SLC25A13, ATP5SL, E2F4, PPIE, GPAM, AP4M1 and ZZEF1, and copy number gains were seen in LPCAT1 and ZFYVE26. Of particular interest was the loss of the E2F4 gene, which encodes a transcription factor and is primarily involved in growth arrest, together with members of the Rb family. E2F4 has also been proposed to play a regulatory role in cellular survival during chemotherapeutic treatment in that it might modulate the predisposition of cells to undergo drug-induced apoptosis [56]. We found >20 interactions between the E2F4 protein known taxane resistance genes. In MDA_SUB-15nM_, we observed copy number gains in the ABCB1 and ADAM22 genes similar to that in the MCF-7_SUB-1.2nM_. The copy number gains observed in these genes were not detected in the final stage (MDA_RES-150nM_), but the fact that the genes were also observed as upregulated in MDA_RES-150nM_ supports the fact that the copy numbers are still present at this stage. In addition, a copy number gain was observed in the JAK2 gene that encodes a tyrosine-protein kinase. Previously, increased expression of JAK2 has been associated with drug resistance in ovarian cancer [57], and differential expression of JAK2 has been associated with taxane resistance in the OC3/TAX300 cell line [58].

For the cell line stages MCF-7_SUB-45nM_ and MDA_SUB-100nM_, a few genomic changes were of special interest. In the MCF-7_SUB-45nM_, these included copy number losses in HTATIP2, involved in induction of apoptosis, and PDCD2L, involved in the cell cycle. In MDA_SUB-100nM_, a missense mutation (chr7:128587374_G/A) was discovered in IRF5 that encodes the interferon regulatory factor 5, a transcription factor involved in the Toll-like receptor signaling pathway. Another member of the interferon regulatory factor family (IRF9), in which we found a copy number gain in MCF-7_SUB-1.2nM_, has been associated with paclitaxel response in that upregulation of IRF9 may be associated with resistance to paclitaxel, and suggested as a potential surrogate marker of response [51].

## Conclusions

In this study, the use of in vitro grown human breast cancer cell lines allowed investigation of genomic alterations during multiple steps in the process towards a docetaxel-resistant phenotype. Variations were prioritized for functional changes as well as those that accumulate over stages of acquiring resistance. A useful extension of this paradigm would be to conduct deeper sequencing in order to examine increasing proportions of resistant sub-clones. In our study, the model system allowed us to identify a number of genomic variations that are consistent with prior knowledge in taxane resistance, and additionally numerous novel genomic variations that were associated with the development of docetaxel resistance. The main conclusion arising from these data is that no single genomic biomarker adequately predicts resistance to docetaxel, but rather requires a panel of markers covering several pathways involved in docetaxel resistance. Specifically, we have identified a number of genetic variations in genes involved in drug transport, microtubule dynamics and cell cycle regulation. A corollary of our observations is that exposure of cells to toxic agents can induce acquisition of stable genomic traits that reduce the effects of these agents, effectively providing a resistance-associated gene pool to tumors. The importance of the disclosed genomic variations - and the fact that docetaxel-resistant cells demonstrated distinct and biologically-relevant DNA aberrations - is that acquisition of docetaxel resistance in clinical breast cancer also is a stable condition that will prevent beneficial effects upon subsequent exposure to the same drug, i.e. repeated docetaxel exposure to patients who relapsed on adjuvant docetaxel treatment. We therefore propose that a set of prioritized findings from our study should be examined at the genomic level in resistant tumors.

## Additional files

Additional file 1: Sheet 1: Summery of acquired genomic alterations being selected for during docetaxel exposure in the MCF-7 sub-lines. Sheet 2: Details on the acquired genomic alterations being selected for during docetaxel exposure in the MCF-7 sub-lines. Positions are annotated with Ensembl VEP (version 2.8). The stage (sub-line) in which the alteration was acquired is given along with assigned variant class. Sheet 3: Same as sheet 2, but showing only *Class A* variation. Sheet 4: Same as sheet 3, but showing only the ‘most severe’ variant per gene. Sheet 5: Summery of acquired genomic alterations being selected for during docetaxel exposure in the MDA-MB-231 sub-lines. Sheet 6: Details on the acquired genomic alterations being selected for during docetaxel exposure in the MDA-MB-231 sub-lines. Positions are annotated with Ensembl VEP (version 2.8). The stage (sub-line) in which the alteration was acquired is given along with assigned variant class. Sheet 7: Same as sheet 6, but showing only *Class A* variation. Sheet 8: Same as sheet 7, but showing only the ‘most severe’ variant per gene. (XLSX 1113 kb) Additional file 2: Sheet 1: A statistic summary of genes from sheet 2. Sheet 2: A list of 396 breast cancer associated genes in which genomic variants were acquired and selected for during docetaxel exposure in the MCF-7 and MDA-MB-321 sub-lines. The variants include *Class A* and *B* variants plus CNA events that correlated with expression changes as observed in MDA_RES-150nM_ and MCF-7_RES-65nM_. The genes are mapped and classified according to the eleven hallmarks of cancer. (XLSX 135 kb) Additional file 3: Sheet 1: Detailed information on the copy number alterations detected in the MCF-7 cell sub-lines. Sheet 2: Detailed information on the copy number alterations detected in the MDA-MB-231 cell sub-lines. Sheet 3: A list of genes in which copy number alterations were detected in the MCF-7 cell sub-lines. A ‘1’ denotes that a CNA is detected in a given stage (sub-line). A ‘0’ indicates that it was not observed. Information on any alterations in expression observed for a gene with a CNA in the final stage (MCF-7_RES-65nM_) is given in last columns. Sheet 4: Similar to sheet 3, but with data for the MDA-MB-231 sub-lines. Sheet 5: Similar to sheet 3 but here showing only those genes in which a CNA has been detected in a given stage (sub-line) of MCF-7 and where the expression of the gene was altered in the final stage (MCF-7_RES-65nM_). Sheet 6: Similar to sheet 5, but with data for the MDA-MB-231 sub-lines. Sheet 7: Overview of the approximate regions in which copy numbers were detected in the MCF-7 cell sub-lines. Sheet 8: Similar to sheet 7, but with data for the MDA-MB-231 sub-lines. (XLSX 1399 kb) Additional file 4: Sheet 1: Results from the GPEC analysis performed across selected genes detected in the MCF-7 sub-lines. Sheet 2: Same as sheet 1, but with data for the MDA-MB-231 sub-lines. (XLSX 73 kb) Additional file 5: Sheet 1: Contains a list of genes included in the GPEC analysis performed on selected genes in each stage (sub-line). Data shown for both cell lines: MCF-7 and MDA-MB-231. Also, a literature-curated list of genes with known association to taxane resistance is given (Ehlers et al, unpublished observations). Sheet 2: Results for the GPEC analysis performed on selected genes in each stage (sub-line) of the MCF-7 cell line. Sheet 3: Same as sheet 2, but with data for the MDA-MB-231 sub-lines. Sheet 4: A data sheet containing the top 10 genes from the GPEC analysis performed on selected genes in each stage (sub-line). Data shown for MCF-7 and MDA-MB-231 sub-lines. (XLSX 106 kb)
